# Comparison of Ma-Griffith combined with a minimally invasive small incision to a modified suture technique for the treatment of acute achilles tendon ruptures

**DOI:** 10.1186/s12891-022-05769-w

**Published:** 2022-08-30

**Authors:** Li Jun, Yu Hao, Zhan Junfeng, Zhang Jisen, Xu Xinzhong, Yao Yunfeng, Tian Dasheng, Xie Jia

**Affiliations:** Department of Orthopedics, The Second Affiliated Hospital of Anhui Medical University, 678 Furong Road, 230601 Anhui Hefei, People’s Republic of China

**Keywords:** Achilles tendon rupture, Minimally invasive surgery, Acute Achilles tendon rupture, Modified MaGriffith method, Modified suture

## Abstract

**Background:**

The Achilles tendon is the strongest tendon in the human body, although it is also prone to injury and rupture. Currently, the best treatment method for acute Achilles tendon rupture remains controversial. The aim of this study was to compare the efficacy of the Ma-Griffith method combined with a minimally invasive small incision (M-G/MISI) with the modified suture technique (MST).

**Methods:**

We conducted a retrospective review of the medical records of all patients who underwent treatment for acute Achilles tendon rupture between January 2012 and January 2020 at our hospital. Demographic characteristics, operative details, and postoperative complications were recorded, and data were statistically analyzed to compare the treatment efficacy of the two operative methods.

**Results:**

A total of 67 patients were enrolled in the study, 34 of whom underwent M-G/MISI treatment, and 33 of whom underwent MST treatment. The intraoperative blood loss in the M-G/MISI group (16.47 ± 13.23 ml) was significantly lower than that in the MST group (34.55 ± 13.01 ml), and the difference was statistically significant (*P* ˂0.001). The incision in the M-G/MISI group (3.79 ± 1.81 cm) was significantly shorter than that in the MST group (5.79 ± 1.00 cm), and the difference was statistically significant (*P*˂0.001). The Achilles tendon rupture score and the American Orthopedic Foot and Ankle Society (AOFAS) score were higher than those of the MST group at the sixth month after the operation (*P*˂0.001). Postoperatively, there was 1 case of traumatic Achilles tendon rupture in the M-G/MISI group and 1 case each of infection and deep vein thrombosis in the modified suture group.

**Conclusions:**

Compared with the MST group, the M-G/MISI group had better Achilles tendon and ankle function scores at 6 months postoperatively, and less bleeding and shorter incisions. M-G/MISI is less invasive than MST.

## Introduction

Although the Achilles tendon is the strongest tendon in the human body, it is one of the tendons that is most prone to injury and rupture [1]. The annual incidence of Achilles tendon rupture is approximately21.5–24.0 cases per 100,000 persons [2]. The mean age at the time of rupture has been reported to be between 30 and 46 years old, with more men than women acquiring an acute Achilles tendon rupture (male-to-female ratio 2.9–5.7:1) [3, 4] Previous studies have suggested that the most common site of injury is 2–6 cm above the calcaneal tubercle [5, 6]. A recent study found that most acute Achilles tendon ruptures occur between 5 and 8 cm above the distal end of the calcaneal attachment point [7]. Acute Achilles tendon rupture requires early diagnosis and treatment; otherwise, it will lead to lower limb dysfunction that has serious impacts on the patients’ quality of life and work, and creates a heavy economic burden for their families [8].

Acute Achilles tendon rupture is treated either by operative or nonoperative (*i.e.,* conservative) methods. Operative treatment generally includes traditional enhanced repair or nonenhanced repair, limited small incision surgery, percutaneous minimally invasive surgery, modified percutaneous repair and the use of special minimally invasive operative equipment for Achilles tendon ruptures, such as an Achilles tendon stapler.

Currently, the best treatment method for acute Achilles tendon rupture remains controversial 9]. Patients with severe underlying diseases who are unwilling to undergo surgery are usually treated conservatively. However, the rerupture rate after nonoperative treatment is high [10]. Compared with nonoperative treatment, surgery can reduce the incidence of Achilles tendon rerupture, although the incidence of other complications related to surgery, such as nerve injury and incision problems, is significantly higher [11–13]. Minimally invasive percutaneous repair is less invasive and can reduce the risk of soft tissue complications [14]. However, because the rupture site of the Achilles tendon is not exposed, some percutaneous repair methods can lead to poor involution of the tear site and weak biomechanical resistance [15].

In light of the problems existing with traditional incision repair and minimally invasive repair, we propose a new operative method: the modified Ma-Griffith method combined with minimally invasive small incision (M-G/MISI) surgery. This method is based on percutaneous suture, limited incision under direct vision, and more reliable sutureing of the ruptured tendon edge of the Achilles tendon. We also used a lumbar puncture needle, which greatly reduced the cost of surgery for patients. Currently, in addition to M-G/MISI, the modified suture technique (MST) is also used to treat Achilles tendon rupture in our hospital. The purpose of this study was to compare and analyze the efficacy of the M-G/MISI with that of the MST for the treatment of acute Achilles tendon rupture.

## Materials and methods

We reviewed and analyzed the medical data of patients who underwent treatment for acute Achilles tendon rupture in the Department of Orthopaedics of the Second Affiliated Hospital of Anhui Medical University of China from January 2012 to January 2020. This study was approved by the local institutional review committee, and the necessary informed consent was obtained.

### Patients

A total of 67 patients were enrolled in the study, 34 of whom underwent M-G/MISI treatment, and 33 of whom underwent MST treatment.

The inclusion criteria were as follows: (1) a diagnosis of acute closed Achilles tendon rupture based on the clinical physical examination and imaging data; (2) age ≥ 18 years old; and (3) the patient having undergone either the M-G/MISI treatment or MST.

The exclusion criteria were (1) open Achilles tendon rupture; (2) chronic pain of the Achilles tendon; (3) calcaneal fracture; (4) old Achilles tendon rupture; or (5) previous local closure of the Achilles tendon.

### Surgery

**Fig. 1:**
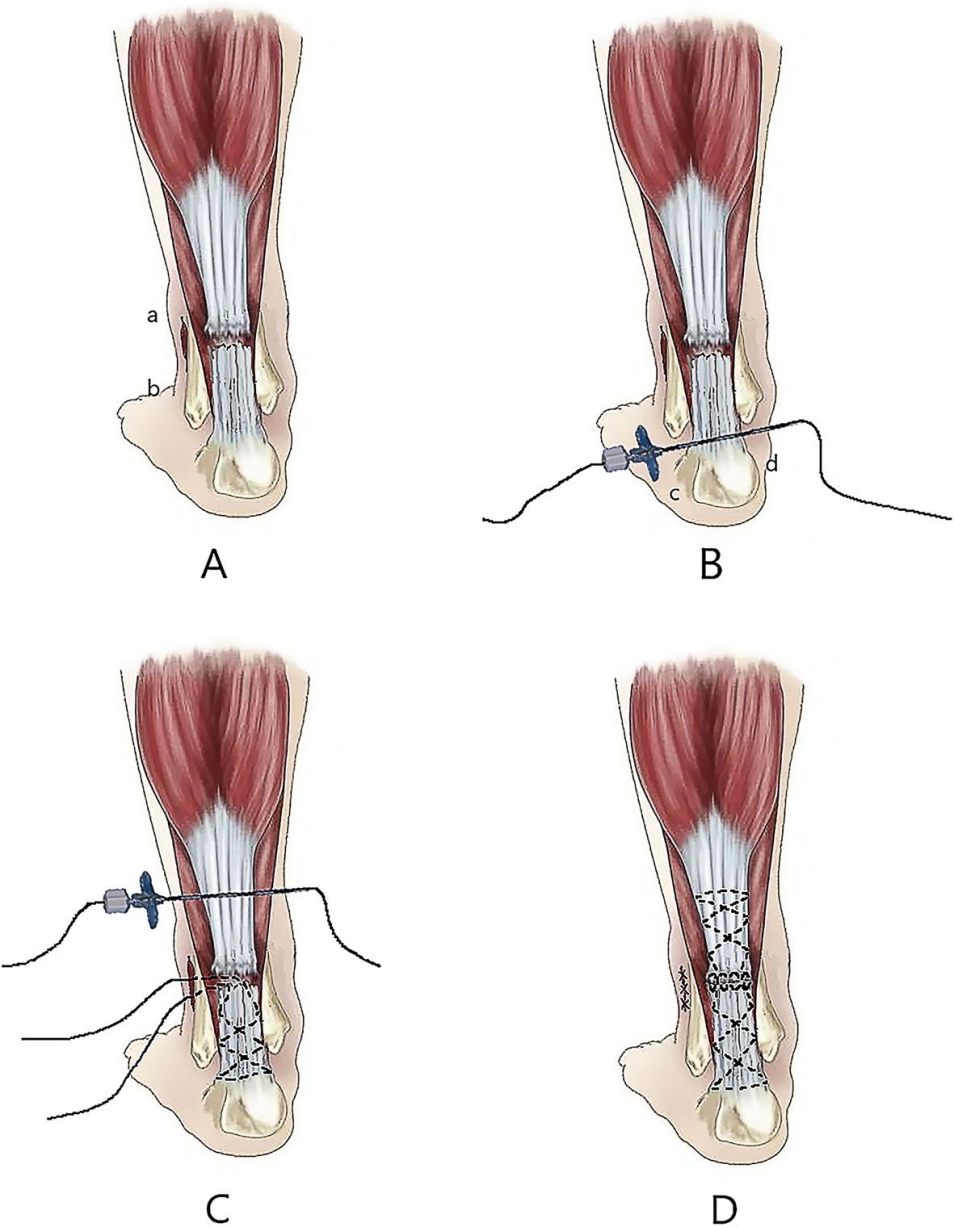
Schematic diagram of the M-G/MISI operation. **A** ab is a small incision on the medial side of the ruptured tendon edge of the Achilles tendon (2 cm left and right, and the incision can be expanded if necessary). **B** cd labels the two symmetrical points of the distal end of the Achilles tendon. Since this area the connection between the Achilles tendon and the calcaneus, the Kirschner needle is used to pass through cd with the help of an electric drill, followed by an epidural puncture needle along the cd through the Achilles tendon, and the PDSIIline passes through it. Then, the epidural puncture needle passes along the dotted line, and the PDSIIline passes through it, the PDSIIline crosses along the ruptured tendon edge in the same way. **C** Symmetrically, the ef point symmetrical to the cd point was found at the proximal end of the ruptured tendon edge of the Achilles tendon. In the same way, the PDSIIline crossed along the ruptured tendon edge and then crossed with the distal PDSIIline to be reinforced and sutured (the black dotted line represents the suture track inside the tendon). **D** When the metatarsal curvature of the affected limb was fixed, after strengthening the suture of the ruptured tendon edge of the Achilles tendon, we checked whether the continuity of the Achilles tendon was good, then rinsed the operation field with a large amount of normal saline, and finally closed the incision

**Fig. 2:**
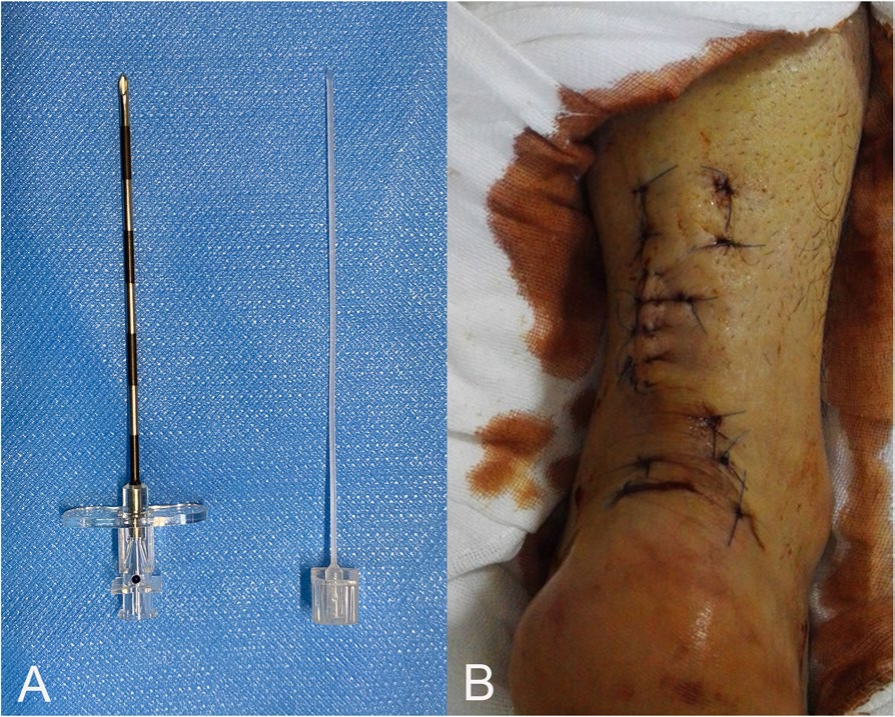
Modified Ma-Griffith method combined with a minimally invasive small incision schematic diagram. **A** Epidural puncture needle; **B** surgical incision photos on the second day after the operation

All operations were performed by several experienced orthopedic surgeons using M-G/MISI (Fig. 1). With the patient in a prone position and under general anesthesia, a 2 to 3 cm incision was made on the medial side of the Achilles tendon (Fig. 1, a to point b). After cutting the skin and subcutaneous and peri-tendon tissue in turn, the hematoma at the ruptured tendon edge was removed, and the ruptured tendon edge of the Achilles tendon was carefully exposed. Tissue forceps were used to organize the ruptured tendon edge and to lengthen the incision, if necessary. The M-G/MISI was used to suture the distal and proximal Achilles tendons with the help of an epidural puncture needle and polydioxanone synthetic absorbable suture (PDS) II line (Fig. 2). At the distal end of the Achilles tendon, a 2 mm Kirschner needle was used to drill holes vertically along the Achilles tendon, and the ruptured tendon edge was pulled with tissue forceps. The Kirschner needle was inserted (Fig. 1 B point c) and drawn out (Fig. 1 B point d) such that the epidural puncture needle was crossed symmetrically through the ruptured tendon edge of the Achilles tendon until the lumbar spinal needle finally pierced the ruptured tendon edge of the Achilles tendon. At the proximal end of the Achilles tendon, the ruptured tendon edge was pulled with tissue forceps, the tendon crossed approximately 3 cm from the ruptured tendon edge, and the epidural puncture needle was inserted and pulled out such that the lines on both sides were equal. Using the same method described above, the epidural puncture needle was with drawn symmetrically at the medial and lateral sides of the ruptured tendon edge of the Achilles tendon. With the ankle in a plantar flexion position, the distal and proximal PDS II lines were tightened. At the ruptured tendon edge of the Achilles tendon with the Fig. 1 ab incision, the knot was fastened from the inside and outside of the Achilles tendon, and then an absorbable suture was used to strengthen the suture at the ruptured tendon edge.

**Fig. 3:**
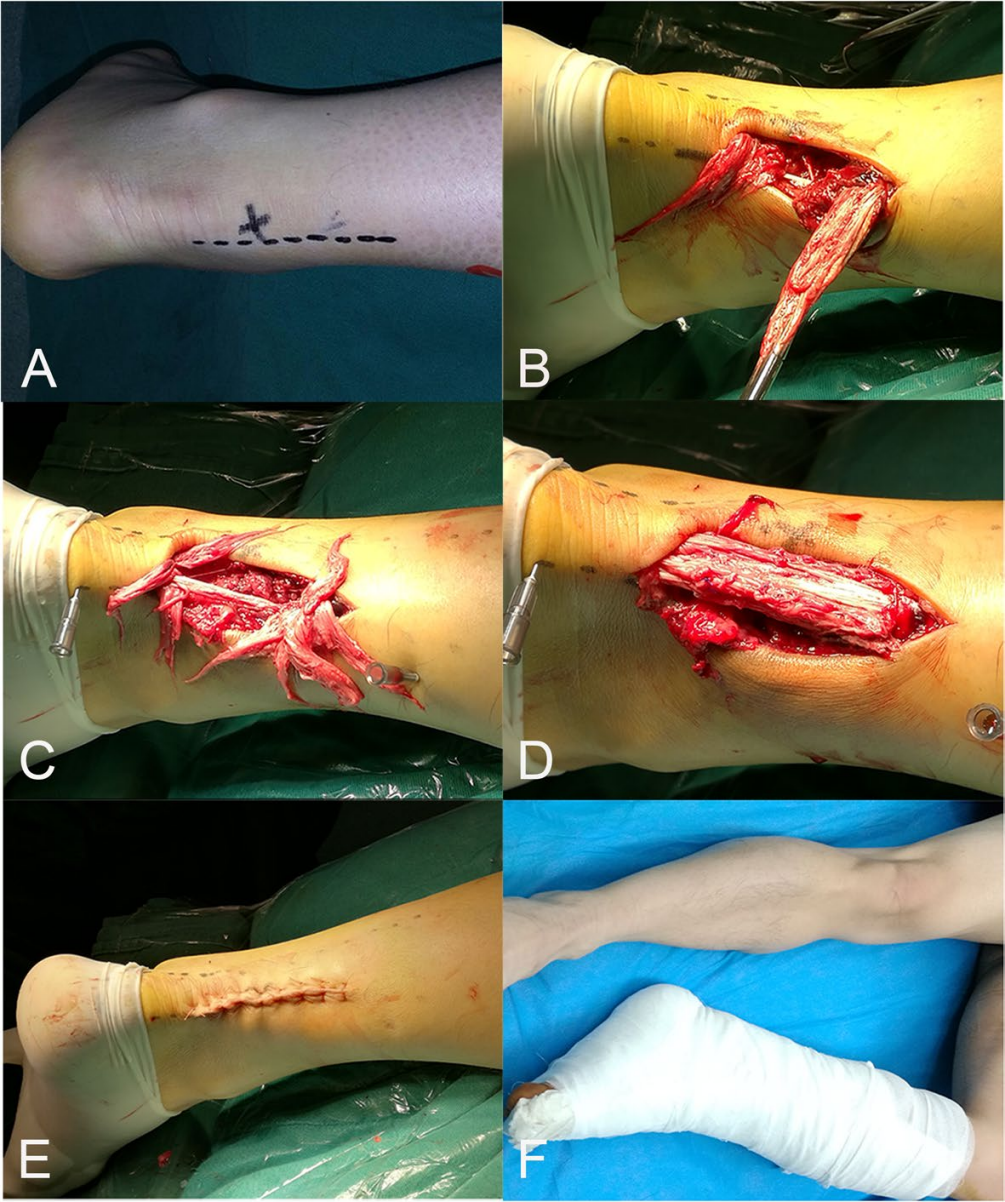
Modified suture treatment schematic diagram. **A** Surgical markings made before the operation. **B** The ruptured tendon edge of the Achilles tendon is a horsetail. **C** The ruptured tendon edge of the Achilles tendon was combed and divided into 4–7 bundles. **D** Suturing of the deep tissue and the ruptured tendon edge of the Achilles tendon. **E** Appearance of the skin after stitching. **F** Postoperative plaster fixation below the knee

Another experienced orthopedic surgeon performed all the of MST treatments (Fig. 3). Under continuous general anesthesia, the patients were placed in the prone position with knee flexion of 30 degrees. Using the ruptured tendon edge of the Achilles tendon as the center, a 5 cm incision was made at the medial edge of the Achilles tendon. The skin was incised to expose the deep fascia and the Achilles tendon’s aponeurosis was cut to the adventitia of the tendon. After exposing the ruptured tendon edge of the Achilles tendon, the blood clot was thoroughly removed, and the ruptured Achilles tendon was lifted, exposing the deep muscle tissue of the tendon. The deep muscle tissue of the Achilles tendon was then sutured with 2–0 absorbable suture, thus building an “Achilles tendon bed” to facilitate the recovery and healing of the Achilles tendon’s blood supply. Then, the caudal calcaneus tendon was combed, and the ruptured tendon edge was divided into 4 to 7 bundles according to the anatomical level of the Achilles tendon. A 5–0 Prolene suture was used to suture one end of the long tendon bundle and the other end of the short tendon bundle vertically and intermittently to maintain the physiological length of the Achilles tendon. To avoid excessive metatarsal flexion of the ankle caused by Achilles tendon shortening, all Achilles tendon bundles were sutured. The adventitia of the Achilles tendon and the tendon aponeurosis were sutured with 3–0 and 2–0 absorbable sutures, respectively. After touching the Achilles tendon to determine good continuity and examining the foot extension to ensure that the ruptured tendon edge of the Achilles tendon was anastomosed firmly, the operative field was rinsed, and the incision was closed layer by layer.

### Postoperative management

After the operation, the affected limb was raised (5-10 cm), and the patient received symptomatic support treatment, including detumescence and analgesia. The ankle joint was fixed at 20-30degrees in the plantar-flexion position with a short leg plaster. After six weeks of immobilization, the cast was removed, and functional exercises were performed on the ankle. Crutch walking was allowed, provided the affected leg was non-weight bearing. Postoperative instructions included functional exercises to strengthen the hip and knee joints, normal flexion and extension of the toes, and active prevention of venous thrombosis of the lower extremities. The details were as follows: exercise of the hip and knee joints within 2 weeks postoperatively; removal of stitches 2 weeks postoperatively; flexion and extension of the toes within 5 weeks postoperatively; removal of plaster immobilization and partial weight bearing with crutches 5 weeks postoperatively; complete weight bearing 9 weeks postoperatively; jogging permitted 12 weeks postoperatively; and patient allowance to resume normal activities 6 months postoperatively.

### Clinical outcomes evaluation

#### Hospital or surgical theater databases

Operative time, surgical incision and blood loss, and length of hospital stay were recorded for a general assessment of surgical complexity and degree of surgical trauma.

#### American orthopedic foot and ankle society score

AOFAS [16] scores were recorded before surgery and at 3 and 6 months after surgery (AOFAS0, AOFAS3, and AOFAS6, respectively). The AOFAS developed four rating systems to provide a standard method of reporting the clinical status of the ankle and foot. The systems incorporate both subjective and objective factors into numerical scales to describe function, alignment, and pain. The maximum score is 100 points (the best possible outcome). Total scores of < 50, 50–74, 75–89, and 90–100 indicate poor, fair, good, and excellent outcomes, respectively.

#### Achilles tendon rupture score

ATRS [17] scores were recorded before surgery and at 3 and 6 months after surgery (ATRS0, ATRS3, and ATRS6, respectively). The ATRS is a patient‐reported instrument with high reliability, validity, and sensitivity for measuring outcomes after treatment in patients with a total Achilles tendon rupture. The ATRS was used to evaluate the limitations of the calf, Achilles tendon, and foot movement after Achilles tendon injury, and systematically evaluated 10 problems such as pain, daily activity, medium‐intensity exercise, and high‐intensity exercise after Achilles tendon injury. The full score of each item was 10, and the degree of functional limitation of the Achilles tendon was classified as slight, moderate, serious, or severe.

#### Complications

Potential postoperative complications, as an indicator of surgical safety, included infection, rerupture, nerve injury and deep venous thrombosis. All complications were recorded. The occurrence of complications affected the safety and feasibility of the operation.

### Statistical analyses

The evaluation consisted of the patient’s sex, affected side, age, operation time, incision length, blood loss during the operation, hospital stay, injury to operation time, AOFAS score, and ATRS. All statistical data were analyzed by specialized statisticians using SPSS software, version 24.0 for Windows (IBM Corp. Released 2016. IBM SPSS Statistics for Windows, Version 24.0). Descriptive data are reported as the means, medians, standard deviations (SDs), and ranges. For statistical testing, a nonparametric test of two samples was used. For all measurements, significance was considered at *P* < 0.05.

## Results

### General data

All 34 patients in the M-G/MISI group were males, and their follow-up time was 22.15±10.29 weeks (ranging from 6 to 50 weeks). In the modified suture treatment group, there were 2 females and 31 males; their follow-up time ranged from 21.24±8.07 weeks (from 9 to 36 weeks). There was no difference in the time of injury between the 2 groups (*P*=0.765). There were no significant difference in the demographic characteristics between the groups. (Table 1)

**Table 1:** Sample characteristics of patients

Group	n	Age	Injury side(L/R)	Gender(F/M)	Smoking	Time from injury to operation (days)
M-G/MISI	34	33.94 ± 7.19	22:12	0:34	26	4.85 ± 1.60
MST	33	36.50 ± 9.00	17:16	2:31	24	4.58 ± 1.84
χ^2^/Z		1.06	1.20	2.12	0.067	0.88
P		0.291	0.274	0.145	0.796	0.765

### Hospital or surgical theater databases

The operative time was 78.12 ± 23.02 min in the M-G/MISI group and 73.73 ± 13.88 min in the MST group, and the difference was not statistically significant (*P* = 0.484). The hospital stay was 9.41 ± 1.46 days in the M-G/MISI group and 9.03 ± 2.42 in the MST group, and the difference was not statistically significant (*P *= 0.241). The blood loss was 16.47 ± 13.23 ml in the M-G/MISI group and 34.55 ± 13.01 ml in the MST group, and the difference was statistically significant (*P*˂0.001). The incision length of patients in the M-G/MISI group was 3.79 ± 1.81 cm (range: 2–6 cm) and that in the MST group was 5.79 ± 1.00 cm (4–8 cm). There were significant differences between the two groups (*P*˂0.001). (Table 2).

**Table 2:** Hospital or surgical theater databases

Group	n	Operation time (min)	Hospital stays (days)	Blood loss (ml)	Length of incision(cm)
M-G/MISI	34	78.12 ± 23.02	9.41 ± 1.46	16.47 ± 13.23	3.79 ± 1.81
MST	33	73.73 ± 13.88	9.03 ± 2.42	34.55 ± 13.01	5.79 ± 1.00
χ^2^/Z		0.70	1.39	5.49	3.89
P		0.484	0.241	0.001	0.001

### American orthopedic foot and ankle society score

The preoperative AOFAS scores of the M-G/MISI group and the MST group were 46.91 ± 5.22 and 47.42 ± 5.02, respectively, and the difference was not statistically significant (*P* = 0.594). The AOFAS scores of the M-G/MISI group and the MST group were 91.21 ± 1.95 and 90.52 ± 1.86, respectively, at 3 months after surgery, with no statistically significant difference (*P* = 0.446). However, the ATRS scores of the M-G/MISI group and the MST group at 6 months after surgery were 97.03 ± 2.71 and 93.18 ± 2.04, respectively, with statistically significant differences (*P*˂0.001)(Table 3).

**Table 3:** PROM scores

Group		AOFAS			ATRS	
	AOFAS0	AOFAS3	AOFAS6	ATRS0	ATRS3	ATRS6
M-G/MISI	46.91 ± 5.22	91.21 ± 1.95	97.03 ± 2.71	47.50 ± 4.96	90.71 ± 2.15	96.71 ± 3.51
MST	47.42 ± 5.02	90.52 ± 1.86	93.18 ± 2.04	46.82 ± 5.13	90.42 ± 1.87	93.18 ± 2.54
|Z|	0.41	1.59	5.11	0.53	0.76	4.09
P	0.685	0.113	0.001	0.594	0.446	0.001

### Achilles tendon rupture score

The preoperative ATRS scores of the M-G/MISI group and the MST group were 46.91 ± 5.22 and 47.42 ± 5.02, respectively, and the difference was not statistically significant (*P* = 0.594). The ATRS scores of the M-G/MISI group and the MST group were 91.21 ± 1.95 and 90.52 ± 1.86, respectively, at 3 months after surgery, with no statistically significant difference (*P* = 0.446). However, the ATRS scores of the M-G/MISI group and the MST group at 6 months after surgery were 96.71 ± 3.51 and 93.18 ± 2.54, respectively, with statistically significant differences (*P*˂0.001) (Table 3).

### Complications

In this study, a similar number of patients in each group returned to their original sporting activities. Of 31 patients who discontinued their sports, 16 (52%) belonged to the M-G/MISI group, and 15 (48%) belonged to the MST group. All patients returned to their previous work, except for 1 patient who required reoperation in the M-G/MISI group as a result of secondary rupture caused by a fall, whereas in the MST group, 1 patient had an infection, and another had a deep venous thrombosis. All 3 patients were treated symptomatically and recovered well after the operation.

## Discussion

The best treatment for AATRs remains a topic of debate, with the options of nonoperative, open surgical, minimally invasive, and percutaneous approaches. The main finding of the present study is that M-G/MISI is superior to MST in the treatment of ankle and Achilles function scores after acute Achilles tendon rupture, and the surgical incision in M-G/MISI is smaller.

Numerous randomized studies have been performed comparing operative versus nonoperative treatment, the Achillon technique to open repair, and various percutaneous techniques to open repair, but its treatment is still debated [18]. Numerous surgical procedures have been proposed to repair Achilles tendon rupture. Open surgery ensures tendon repair and improves healing, thus leading to a lower rerupture rate (approximately 2–5%) [19], but complications are common, in up to 34% of cases, including wound infection, skin binding, sural nerve injury, and hypertrophic scarring [20]. Studies have shown that although open repair is the most common surgical technique, surgeons have significant differences in surgical and suture techniques [21]. Percutaneous and minimally invasive techniques combining the benefits of conservatism and openness, such as Ma and Griffith (M&G) or Tenolig, are believed to reduce the risk of complications. Open surgery usually requires a longer incision (approximately 10 cm on average) and too much shedding of the Achilles tendon tissue, which can affect postoperative recovery [22], and large incisions lead to excessive intraoperative blood loss. However, in this study, the average incision length of M-G/MISI was only 3.79 cm (1.81), which was significantly shorter than that with the MST. In a previous randomized controlled study, the clinical and functional scores of patients in the open surgery and percutaneous repair groups were similar, with no significant difference in the incidence of complications between the two groups [23]. However, subsequent studies have found that percutaneous repair of acute Achilles tendon ruptures resulted in fewer complications and higher recovery of activity levels. In our study, there was 1 case of traumatic Achilles tendon rupture in the M-G/MISI group and 1 case each of infection and deep vein thrombosis in the modified suture group. The above results indicate that shorter surgical incisions can effectively reduce intraoperative blood loss and reduce the risk of postoperative infection. Patients undergoing minimally invasive percutaneous surgery generally have a shorter operation time [24], but our results were different and there was no significant difference in the operation time between the two groups. This finding might have been due to the learning curve of surgery, or the number of cases that we included was not sufficient.

Studies have found that compared with minimally invasive surgery, traditional incision repair is more stable but also more prone to infection [25]. In a retrospective analysis of 4,477 patients, Popovic et al. reported that the total incidence of complications was 12.5%; the most common complication was mild incision problems (6.5%), and the rerupture rate was 1.5% [26]. In fact, the problem of incision complications was considered as early as 1977, when Ma-Griffith proposed the Ma-Griffith method [27]. Although this method can reduce the risk of infection and avoid long incisions, the strength of the percutaneous suture has been questioned. Some researchers have studied the method of surgery and found that the rate of rerupture was as high as 8%, and the rate of sural nerve injury was as high as 19% [28]. In comparison with open surgery [29], there were significantly fewer main complications, especially necrosis, in the percutaneous repair group than in the incision repair group, and the total number of complications was smaller.

Adequate repair of Achilles tendon rupture is key to the recovery of ankle joint function. In a recent prospective, randomized clinical trial, the efficacy of open surgery, minimally invasive surgery, and nonoperative treatment for acute Achilles tendon rupture was compared [30]. There were no significant differences in American Orthopedic Foot and Ankle Society ankle-Hindfoot scores among the three treatments at a 24-month follow-up of 69 patients [30]. Different from this RTC, this study used Ma-Griffith combined with a minimally invasive small incision. The results showed that AOFAS and ATRS scores were better in the M-G/MISI group at 6 months after surgery, indicating that the M-G/MISI group had better ankle function recovery.

Sural nerve injury is an important complication of percutaneous minimally invasive surgery. The incidence of sural nerve injury varies with different suture techniques. Lo et al. found that the rate of sural nerve injury in 701 patients was 6.0% [31]. According to different reports, percutaneous repair techniques have different rates of sural nerve injury [32–34]. When using the percutaneous puncture technique to repair the Achilles tendon, sural nerve injury is inevitable because it cannot be sutured under direct vision. In 2002, Assal et al*.* conducted a prospective multicenter study using a limited incision and a specially designed device, the Achillon stapler, to ensure that all sutures were placed into the peritendinous tissue, thereby minimizing the risk of implanting suture material into the sural nerve; none of the patients in that study experienced nerve injury [35]. However, this type of operation requires expensive instruments with limited open repair, which placing a significant financial burden on patients. In this study, the reasons for the absence of sural nerve injury in the M-G/MISI group could be the following. First, the surgeons in the M-G/MISI group were familiar with the location of the sural nerve. Second, the sample size of this study was small; therefore, the complication results do not fully reflect the risk of sural nerve injury. No sural nerve injury was found in the MST group, which could be due to the larger surgical incision and less compression or injury of the sural nerve during the operation. Furthermore, the two surgical methods do not use expensive instruments; thus, the cost is not high. Obviously, patients in the M-G/MISI group lost less blood during the operation because of the smaller incision and less damage to the soft tissues during the operation. The possible reasons for the difference in AOFAS and ATRS scores between the two groups six months postoperatively are as follows. First, the MST involves only suturing of the long bundle and short bundle at the ruptured tendon edge, and the contact surface of Achilles tendon recovery in the M-G/MISI group was larger than that with the MST. Second, since the patients were followed up mainly on an outpatient basis, the AOFAS and ATRS scores in the two groups were provided by the two groups of surgeons; thus, subjective factors cannot be excluded.

There are some limitations in this study. Selection bias was not avoided, considering that the surgical treatment to be performed was determined by the orthopedic surgeon. Confounders, such as BMI and underlying medical conditions, were not completely excluded.

## Conclusions

In conclusion, this study found that, compared with the MST group, the length of the incision in the M-G/MISI group was smaller, the amount of intraoperative blood loss was less, and the ankle and Achilles tendon function scores for a certain period of time were better than those in the MST group. Therefore, M-G/MISI is less invasive than MST, and the functional score of the M-G/MISI group was better than that of the MST group at the sixth month after operation.

## Data Availability

All data generated or analyzed during this study are included in this published article.
